# Biopolymer Coatings for Biomedical Applications

**DOI:** 10.3390/polym12123061

**Published:** 2020-12-21

**Authors:** A. Joseph Nathanael, Tae Hwan Oh

**Affiliations:** School of Chemical Engineering, Yeungnam University, Gyeongsan 38541, Korea

**Keywords:** biopolymers, bioactivity, coatings, biomedical applications, tissue engineering, nanoparticles

## Abstract

Biopolymer coatings exhibit outstanding potential in various biomedical applications, due to their flexible functionalization. In this review, we have discussed the latest developments in biopolymer coatings on various substrates and nanoparticles for improved tissue engineering and drug delivery applications, and summarized the latest research advancements. Polymer coatings are used to modify surface properties to satisfy certain requirements or include additional functionalities for different biomedical applications. Additionally, polymer coatings with different inorganic ions may facilitate different functionalities, such as cell proliferation, tissue growth, repair, and delivery of biomolecules, such as growth factors, active molecules, antimicrobial agents, and drugs. This review primarily focuses on specific polymers for coating applications and different polymer coatings for increased functionalization. We aim to provide broad overview of latest developments in the various kind of biopolymer coatings for biomedical applications, in order to highlight the most important results in the literatures, and to offer a potential outline for impending progress and perspective. Some key polymer coatings were discussed in detail. Further, the use of polymer coatings on nanomaterials for biomedical applications has also been discussed, and the latest research results have been reported.

## 1. Introduction

Tissue engineering research is the combination of materials science, engineering, chemistry, biology, and medicine; hence, it can be considered an interdisciplinary scientific research field. The regeneration of adult tissue following an injury or degeneration is quiet a limited process. Although promising results have been observed in certain laboratory studies, clinical trials are limited, due to certain hindrances posed by cell integration, migration, and survival. Hence, in tissue engineering, the optimization of biointerfaces, their integration with the corresponding tissues, and dominance over their properties are the key guidelines for the development of novel materials. A wide range of polymers can be used in biomedical applications, and there are numerous approaches for utilizing polymers in medical implants and devices. In the biomedical field, synthetic biodegradable polymers find applications in various fields. Biopolymers exhibit good bioactivity, bioresorbability, and nontoxicity. A detailed understanding of biopolymer structure and properties can increase the applicability of biopolymers in various medical processes and improve their use as promising and versatile candidates for coatings in such applications.

In tissue engineering, it is important to provide appropriate biointerfaces, understand the process of their integration with tissues, and control their properties. This control is especially important in the construction of drug or biomolecule delivery systems. Control over the delivery system can be achieved using suitable combinations of polymers and bioactive molecules. Over the past decades, the importance of polymer matrices in hybrid biomaterials has been established based on the wide variety of choices available, ranging from synthetic to natural biopolymers with a good combination of flexibility, biodegradability, adjustable mechanical properties, bioactivity, and low toxicity. Further, polymeric hybrid materials can be prepared as “smart” materials, using which functionality can be achieved through physical, chemical, or biological stimuli.

Polymer coatings are increasingly popular in various diverse applications and segments. From simple coatings to nanoparticle incorporated functionalized composite coatings, these polymer coatings provide a strong functionalities to their host materials. It can be applied in various materials of choice such as metals, ceramics, polymers and nanoparticles. In biomedical field, polymeric coatings can play a vital role for the development of next generation biomaterials and instruments. They can be applied as corrosion resistance, functionalize the surface, wear resistance, improve bioactivity and even can be used as a switchable smart materials. Smart polymer coatings are the recent advancement in the polymer coatings. Various reports indicated that, polymer are smart and have the capability to respond to numerous stimuli, such as temperature, light, magnetic field, electric field and pH [1]. In the medical field, these smart polymers are mainly used in drug delivery applications where the drugs can be loaded in these polymer or polymer coatings and can be delivered at the chosen location with the aid of a stimuli. Further, shape memory polymers are gaining interest and are promising candidates for bone tissue engineering applications.

Another interesting aspect of the polymer coatings is the formation of the composite with other polymers or inorganic compounds. The development of polymer-based composites are one of the main approaches in resolving the glitches associated with the polymers in biomedical applications. Balanced physical, chemical and biological properties can be achieved through blending the polymers with other materials. After the emergence of nanotechnology, nanoparticle incorporation in with polymer base materials provide improved functional characteristics. In drug delivery field, polymer based nanocarriers are very promising owing to their ability for the encapsulation of drugs, controlled delivery, sustained release and bioactivity. By incorporating suitable nanofillers, polymer nanocomposites can be designed for versatile applications.

This review aims to offer an overview of the latest progress in the biopolymer coatings and their applications in various biomedical field, to highlight the most important results in the literature, and to offer a potential outline for impending progress and perspective. Initially, brief overview of the polymer coatings and their methods are described. Then, the short survey on the polymer coatings on metal surfaces are described. Further, various biopolymer coatings and their application in the biomedical fields are described separately. Finally, the application of biopolymer coatings on nanoparticles are discussed. There are numerous reports available for biopolymer coatings and various polymers are used for biomedical coatings. In this review, very recent research reports are taken into account and some specific biopolymers are given emphasis based on their wide application and coating ability in biomedical field along with some standout reports are provided.

## 2. Polymer Coatings and Films

Polymer coating is an approach for surface property modification undertaken to satisfy the requirements of multiple practical applications. It is a coating or paint produced using polymers with better properties than existing ones. Polymer coatings have been used in various applications, such as adhesion, scratch and abrasion resistance, corrosion resistance, wettability, and bioactivity. Polymer coatings are considered highly useful in biomedical applications because they provide flexibility with respect to the chemical groups that can be attached to surfaces, which are beneficial for biomaterial and tissue interactions. Furthermore, their mechanical and elastic properties are comparable to those of biological tissues. Various methods have been established and implemented for the fabrication of polymer coatings for different applications. Highly efficient coatings with advanced properties can be produced through a prudent choice of material, coating methods, and production parameters. The inherent surface properties of polymers, such as poor wettability and low surface area, lead to substandard bioactivity and make it challenging to use these in implants. Conversely, polymer-coated implants can serve as biomimetic surfaces in the body [2]. Polymers can also be used to coat the surface of nanoparticles to improve their performance in the delivery of biomolecules and drugs. Polymer coatings can be used to improve both hardness adjustment and component delivery.

## 3. Biopolymer Coating Methods

There are various biopolymer coating materials available, which are well-documented in the literature. Since this review specifically focuses on polymer coatings, this section briefly discusses some of the main coating methods used for biopolymer coatings. The assembly of polymer into coatings and films can be done various methods such as layer by layer (LBL) [3,4], polymer brushes, dip coating, Langmuir-Blodgett (LB) [4,5,6], plasma based coating methods, spin coatings and hydrogels. In LBL method, positively and negatively charged polyelectrolytes are coated successively. There are number of charged polyelectrolytes are available to produce LBL film and coatings. The biggest advantage of the LBL process is its flexibility to produce the polymer coatings. Recently Landry et al. reviewed self-assembled layers and multilayer polymer coatings for tissue engineering applications [3]. LBL polymer films as drug delivery carriers was reviewed by Park et al. [4]. LB films are often considered as alternate for LBL process in which the substrates are drawing at a steadily at a constant speed from the polymer solutions. In LBL, molecules from the bulk solution are coated on the substrate, whereas in LB, molecules from the solution surface are coated on the substrates. Leontidis described about LB films and compared it with LBL method [5]. Polymer brush is another popular and interesting surface modification technique in which soft material is covalently tethered on the surface of the substrate [7] and has potential applications in various fields. Various researchers have reviewed the applications of polymer brushes in biomedical field [7,8,9]. Plasma based polymer coatings (especially non-thermal plasmas) are enable us to have controlled polymer coatings on any substrate surfaces for various applications. This coating method provides strong adhesion to extensive range of substrates, such as metals and ceramics. Further, this plasma coatings allows to coat even complex shapes. Plasma based polymer coatings were reviewed and reported by various researchers [10,11,12]. Similarly, various other methods such as dip coating and spin coatings are used for polymer coatings. These methods are simple, cost effective and the coating parameters can be changed easily. In a dip coating process, the substrate is dipped into a polymer coating solution and kept in this solution for some times, which allow substrate to absorb the polymer molecules. After that, the substrate is withdraw from the solution and dried. Evan a large substrates can be uniformly coated by this dip coating process. Various parameters such as solution viscosity, dipping time, drawing speed and drying atmosphere. In spin coating, polymer solution is added dropwise in the center of the substrate, which may be either still or set in to rotate in low speed. After that, the rotation of the substrate is increased to high velocity to facilitate homogenous spreading of the polymer solutions on the substrates with the combined effect of centrifugal force and surface tension. Here, the thickness of the coating was achieved by rotation speed, viscosity and surface tension. Very recently, Song et al. reviewed various biopolymer based coating methods [13].

## 4. Biopolymer Coatings on Metal Implants

In multiple industrial applications, metal surfaces coated with organic layers provide numerous advanced features with high potential for applicability. This technique permits the tailoring of various characteristics, such as elasticity, wettability, bioactivity, and adhesiveness [14]. Biodegradable polymer coatings can be applied as corrosion resistance coatings in implants to prevent corrosion post implantation [2].

Improved bioactivity, corrosion resistance, and antifouling properties were achieved for 316L stainless steel (SS) by applying a pseudopeptide polymer coating. Liu et al. [15] used poly(2-methyl-2-oxazoline) (PMOXA) as a pseudopeptide polymer to produce a non-brush bionic polymer coating by electrochemical assembly on a 316L SS surface. bioactivity and anti-fouling properties were observed for PMOXA with a modest degree of hydrolysis and molecular weight. Further, they found that cell migration and proliferation were enhanced by these coatings. It was claimed that the coating could successfully modify the surface of the complex 3D vascular stent, which could have potential applicability in the prevention of late stent thrombosis and in-stent restenosis [15].

Gnedenkov et al. suggested a unique method for producing composite polymer coatings on the surface of the magnesium alloy MA8 [16]. Significant improvements were reported in the protective and antifriction properties of the magnesium alloy surfaces owing to the special treatment of plasma electrolytic oxidation (PEO) coatings by super-dispersed polytetrafluoroethylene (SPTFE). Further, the authors inferred that the thickness of the composite coating increased due to SPTFE treatment of the PEO coating. The same group reported a similar PEO-based method for preparing hydroxyapatite- polytetrafluoroethylene (PTFE) composite coatings on Mg–Mn–Ce alloys for use in resorbable implants [17]. In this study, the elemental composition, phase, morphology, and multifunctional corrosion resistance of the composite coatings were reported, and these were confirmed to impart bioactivity to magnesium implants.

Oosterbeek et al. attempted to produce polymer-bioceramic composite coatings on magnesium to improve the corrosion resistance of magnesium to increase the degradation time [18]. The composite coating was produced through a two-step process: Initial bioceramic coating by immersion in a supersaturated calcium phosphate solution, followed by polylactic acid coating using a dip coating process. Mechanical testing of this composite coating revealed that the polymer-only coating showed greater adhesion strength compared to the polymer-bioceramic composite coating owing to the poor bonding between the bioceramic layer and the substrate. The presence of corrosion resistance also indicated that the polymer-only coating had a longer period than the other composite coatings.

Recently, similar studies on conferring corrosion resistance and inducing biological properties in magnesium alloys for implant applications were successfully implemented using calcium phosphate/collagen (CaP/Col) composite coatings [19]. Chemical conversion and dip-coating methods were adapted to fabricate composite coatings. The CaP/Col coating effectively reduced the degradation rate of magnesium alloys and increased osteoblast adhesion in the optimal microenvironment and interface created.

In the following section, we have reviewed specific biopolymer coatings as well as the coatings that can be used to modify these polymer surfaces for better utilization in specific biomedical applications.

## 5. Biopolymer Coatings for Surface Modification

### 5.1. Polyvinylidene Fluoride (PVDF)

PVDF is a polymer used widely in medical applications and has been studied extensively. It possesses enhanced biological, textile, and piezoelectric properties, and it is a highly non-reactive thermoplastic fluoropolymer [20,21]. Surgical meshes and sutures require non-reactivity, whereas wound-healing applications require piezoelectricity [21]. Hence, this material is considered suitable for various biomedical applications such as tissue engineering [22,23,24,25,26,27], physiological signal detection [28,29,30,31,32], and antimicrobial and antifouling material development [22,33,34,35,36,37,38]. However, it is difficult to produce coatings for biomedical applications using PVDF because it does not form smooth films and exhibits issues in adhesion with other substrates. There have been reports of effective coating using PVDF and its copolymers using the spin coating and Langmuir–Blodgett (LB) methods. Yin et al. reviewed studies reporting the application of PVDF and its copolymer films using the spin coating and LB methods [39]. Several studies have reported that PVDF, along with other materials, form a composite or combination of materials that offer the advantages of both materials [22,23,24,28,29,31,33,35,36,37,38,39].

Tien et al. [28] fabricated a flexible electronic skin (e-skin) that mimics the functions of the human finger. The device was designed on top of a flexible platform with an array of pressure and temperature sensor pixels, which could be used as a channel using an organic semiconductor (pentacene). The gate dielectric material was produced from a mixture of poly(vinylidenefluoride-trifluoroethylene) (P(VDF-TrFE)) and BaTiO_3_ nanoparticles. This device showed high sensitivity and could be used successfully as e-skin. Hybrid ZnO nanoneedles and PVDF films were fabricated for applications in wireless real-time pressure sensors to monitor the heart rate. These were highly sensitive and wearable, and could detect pressure as low as 4 Pa [31]. A similar type of temperature-sensing material with high thermal responsivity, stability, and reproducibility was constructed by Trung et al. [40]. This temperature sensor used reduced graphene oxide (R-GO) instead of BaTiO_3_ and formed a nanocomposite with P(VDF-TrFE, (R-GO/P(VDF-TrFE)), which acted as a sensing layer. This sensor was reported to be mechanically flexible, optically transparent, and highly responsive to temperature changes (could detect temperature changes as low as 0.1 °C).

**Figure 1:**
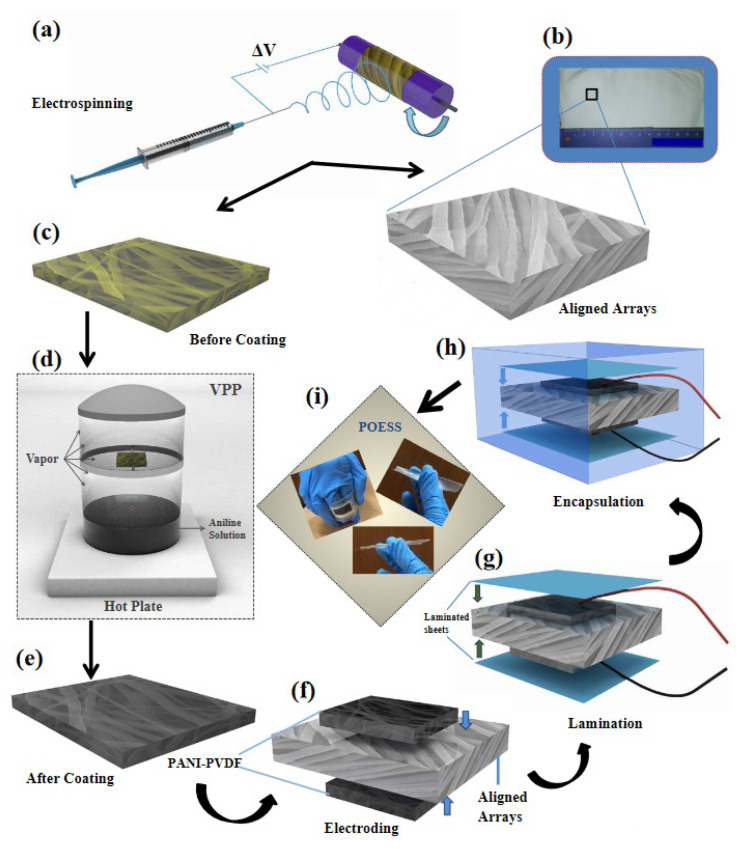
(**a**) electrospinning process, (**b**) photograph of the large-scale prepared mat of highly aligned PVDF NFs arrays; enlarged view exhibits the structure from respective section, (**c**) structure of oxidant-contained yellowish PVDF NFs mat before PANI coating, (**d**) VPP process, (**e**) structure of deepbluish PVDF NFs mat after PANI coating, (**f**) electrode assembling, (**g**) lamination process, (**h**) PDMS encapsulation of POESS design, (**i**) photographs of POESS with demonstration of flexibility. Schematic illustration of the piezo-organic-e-skin sensor design architecture. (Reprinted with permission from [45] Copyright (2020), American Chemical Society.).

Apart from the PVDF coating, electrospinning of PVDF, and its copolymers and composites with different nanomaterials can be performed to utilize advantages of both materials. These composites are mostly used in energy harvesting and environmental remediation applications [41,42,43,44]. In recent times, only a limited number of reports have been published on their applications in the biomedical field. In a recent report, a self-powered piezo-organic-e-skin sensor was constructed using highly aligned PVDF nanofibers as piezoelectric active components and polyaniline-coated PVDF NF mats as flexible electrodes (Figure 1) [45]. This sensor was used for human health monitoring, and exhibited remarkable sensitivity to human finger touch (10 V under 10 kPa) by converting mechanical energy into electric energy. It was also designed to monitor various human gestures such as bending, stretching, compression, movement, coughing, and swallowing.

Electrospun PVDF-nanosilica scaffolds were prepared, and their mechanical and piezoelectric properties were studied for biomedical applications by Haddadi et al. [46]. Hydrophilic and hydrophobic silica nanoparticles were used to construct the composite fiber, and the average fiber diameter was increased by nanoparticle addition. Hydrophilic silica nanoparticles showed higher tensile strength compared to other fibers owing to their higher dispersion and compatibility. The piezoelectric property was enhanced upon the addition of silica nanoparticles; however, the addition of hydrophilic silica led to an increase in the output voltage [46].

**Figure 2:**
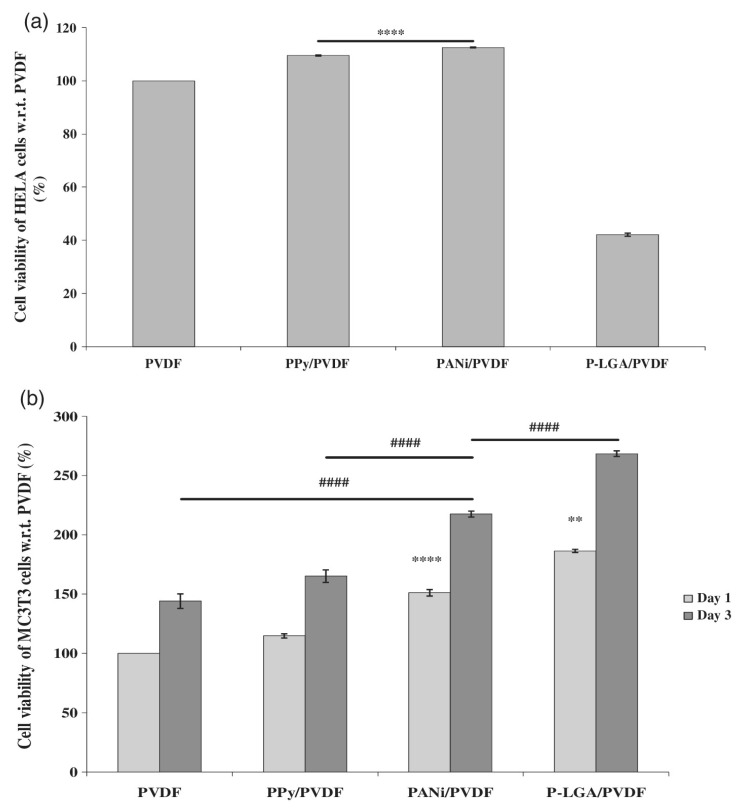
Viability of (**a**) HeLa cells and (**b**) MC3T3 cells on PVDF and PVDF:CP electrospun fibers (control: PVDF). ** and **** signifies *p* < 0.01 (1d) and *p* < 0.0001 (1d), respectively, for both HeLa and MC3T3 culture (1d); #### signifies *p* < 0.0001 (3d). CP, conducting polymers; PVDF, poly(vinylidene fluoride) (Reprinted with permission from [47] Copyright (2020), John Wiley and Sons.)

The blending of PVDF with a conducting polymer is a method for increasing electrical output from PVDF. Sengupta et al. [47] prepared PVDF blends with various polymers (polypyrrole (PPy), polyaniline (PANI), and a modified PANI with L-glutamic acid (referred to as PANILGA/P-LGA)) to obtain different electrically active membranes. Bioactivity, electrical conductivity, β-phase content, and the nanostructures formed were analyzed, and bioactivity was observed to decrease in the following order: p-LGA/PVDF > PANI/PVDF > PPy/PVDF > PVDF. Furthermore, while P-LGA/PVDF exhibited higher bioactivity and electrical conductivity, it also exhibited high cytotoxicity toward HeLa (cancer) cells (Figure 2). Hence, this composite material can be of interest in certain specific biomedical applications.

Wang et al. [48] described the optimized electrospinning conditions for P(VDF-TrFE) nanofiber formation and its effect on the piezoelectricity of P(VDF-TrFE). This nanofiber scaffold was then implanted in SD rats as energy harvesters, and cell proliferation and cell alignment growth were studied. The P(VDF-TrFE) implant provided a maximum voltage and current of 6 mV and 6 nA, respectively. Furthermore, the fibroblasts proliferated and aligned seamlessly along the electrospinning direction of the nanofibers, and proliferation was observed to be enhanced by 1.6-fold. Based on this, the authors claimed that the P(VDF-TrFE) scaffold could be used as a suitable tissue engineering and wound healing material [48].

### 5.2. Polymethyl Methacrylate (PMMA)

PMMA is a synthetic polymer that is lightweight, cost effective, easy to manipulate, and contains harmless subunits; these properties make it suitable for usage in biomedical applications. It is broadly used for various medical applications, such as drug delivery, as well as in tools such as bone cement and microsensors [49]. PMMA is also the material of choice for the production of denture base, orthodontic retainers, and repair in dentistry [49,50]. It exhibits good mechanical properties, slow degradation, low toxicity, and inertness. Due to these properties, it is widely used in hip-joint transplantation. Its non-biodegradable nature makes it suitable for the construction of permanent and mechanically stable structures, such as those used in bone tissue engineering [49,51]. The issues associated with the coating of organic materials on metal surfaces include poor adhesion between these two components. To address this, polymers can be covalently anchored to the substrate surface to generate an adhesive interlayer.

**Figure 3:**
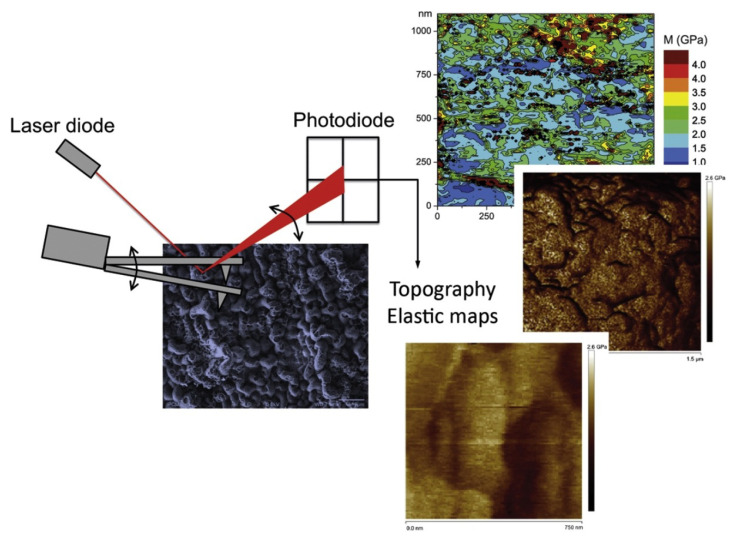
Multiscale mechanical analysis of polymethyl methacrylate layers grafted on Ti substrates. (Reprinted with permission from [14] Copyright (2017), Elsevier).

A 1–2-µm PMMA layer was incorporated on a Ti substrate through alkali activation of the surface [52]. This was achieved through the initial alkali activation of the Ti substrate followed by surface-initiated atom transfer radical polymerization. This was performed in a heated NaOH solution. This treatment produced a porous Ti layer rich in hydroxyl groups. Next, using phosphonic acid as the coupling agent, the polymerization initiator was covalently grafted onto the surface [53]. The coating was approximately of 1.9 µm and was stable in a simulated body solution. Additionally, it exhibited good bioactivity [52]. This method can pave the way for hybrid prosthesis using personalized medicine. In another study, the same method was used to develop a TI/PMMA/Ti sandwich structure and study their adhesion and formability. A high bonding strength and optimal formability were achieved. The results showed that there was no failure or delamination between the Ti and PMMA interfaces. Hence, this type of coating and adhesion method will be advantageous in future biomedical applications. Furthermore, the same authors reported the production of a hybrid Ti/PMMA-layered material and analyzed multiple mechanical characterizations using the same [14]. The mechanical characterization of the thick PMMA layers on Ti substrates was performed using nanoindentation as well as different atomic force microscopy techniques. Each of these methods indicates the mechanical properties at different scales (Figure 3).

Biomaterial modelling for optimized methacrylate coating for Ti implant was proposed by Sun et al. [54]. They applied cheminformatics methods to methacrylated proteins to estimate their suitability as Ti implants coatings. They found that the bioactivity of Ti implants was higher than that of uncoated samples when methacrylated proteins such as GelMA were used. In addition, this coating was less susceptible to biofilm formation, which reduced the risk of osteomyelitis, which ultimately leads to implant fixation. The development of hybrid nanoparticle coating derived from bio-polymer was reported by Galvão et al. [55]. PMMA nanoparticles were synthesized in the presence of poly(diallyldimethyl ammonium) chloride (PDDA) by emulsion polymerization. The antimicrobial coating was created by spin coating or casting and drying of the nanoparticle dispersion using different substrates such as Si, glass, or polystyrene sheets. At its highest relative content, PDD:PMMA mostly produced homogeneous coatings. The presence of PDDA in the coatings significantly inhibited bacterial activity, which was tested in *Escherichia coli* and *Staphylococcus aureus*. The coatings were suggested to be suitable for different biomedical applications.

**Figure 4:**
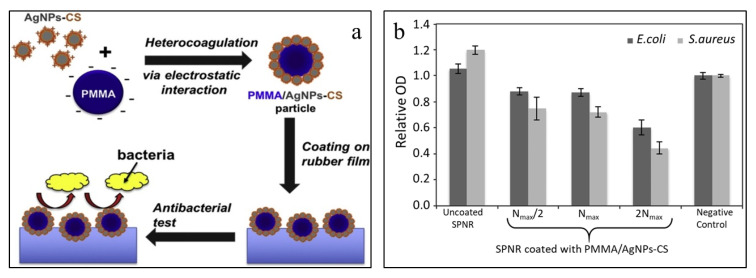
(**a**) Schematic illustration of polymethyl methacrylate/chitosan-silver (PMMA/AgNPs-CS) coating and its antibacterial activity; (**b**) optical density of suspension of *Escherichia coli* and *Staphylococcus aureus*. (Reprinted with permission from [56] Copyright (2019), Elsevier).

The deposition of PMMA/chitosan-silver (PMMA/AgNPs-CS) nanoparticles on a soft rubber substrate was achieved by immersion method which improved antibacterial activity [56]. Positively charged AgNPs-CS (38 nm) was heterocoagulated on the negatively charged PMMA cores (496 nm) to produce PMMA/AgNPs-CS, which was then deposited on the rubber substrate. Antibacterial activity toward *E. coli* and *S. aureus* was amplified on the coating surface (Figure 4). Furthermore, the cytotoxicity of L-929 fibroblast cells was also reduced by these coatings. However, inhibition of the L-929 fibroblast cells was not observed. This study showed that these types of coatings can be applied to various soft substrates in different biomedical applications.

### 5.3. Polypropylene (PP)

PP has been widely used in medical applications, especially as a surgical mesh to strengthen weakened tissues. PP is a thermoplastic polymer with different densities and can be classified into copolymer and homopolymer components. In the biomedical field, PP mesh has been extensively used in urogynecology [57] and hernia repair owing to specific characteristics, such as inertness, hydrophobicity, and strong mechanical properties, even while being lightweight [58]. It also finds applications in other areas of medicine such as breast reconstruction or as a supportive soft tissue structure and blood oxygenator membrane. In addition, it exhibits low potential of carcinogenesis in the human body [59]. However, certain complications are also associated with its use, including the induction of infections and inflammatory responses within the body, which lead to a slow healing process, insufficient drug absorption, and immune system response. Due to its high hydrophobicity, it exerts adverse effects such as tissue damage and insertion resistance in the human body occasionally. Hence, even though PP is a good material, its application is limited owing to its poor biological properties. Therefore, to use PP in medical applications in the human body, surface treatment for improving bioactivity is necessary.

Recently, Saitaer et al. [58] reported the surface modification of a PP hernia mesh using polydopamine (PDA) modified with cold oxygen plasma. This modification led to improved drug absorption and longer release, and also imparted antibacterial properties. Plasma treatment is one of the best methods conventionally used for surface modification for inducing chemical functionality and surface charge and for increasing surface hydrophilicity [60]. Plasma-enhanced chemical vapor deposition (PECVD) was used to modify the surface of PP implants with different chemicals to produce charged PP substrates for layer-by-layer (LBL) coatings [60]. This PECVD method increased hydrophilicity and the number of functional reactive groups available for molecule grafting, and was found to be suitable for LBL deposition on PP substrates.

To tackle the high hydrophobicity of the PP surface, Jang et al. [61] developed a matrix combining polyvinyl pyrrolidone (PVP) and cross-linked polyethyleneglycolacrylate (PEGDA) to produce a stable hydrogel forming layer (PVP:PEGDA) that exhibited hydrophilicity and bioactivity. This study revealed that the hydrophilic nature of the film improved, and the mechanical as well as adhesive strength of the PP surface could be optimized by adjusting the PVP and PEGDA ratio. Compared to the PVP film, the combination of the PVP:PEGDA films showed 7-fold higher tensile strain at the breaking point, and 54-fold higher adhesion strength, respectively. This type of surface modification can be useful for the development of PP medical products.

**Figure 5:**
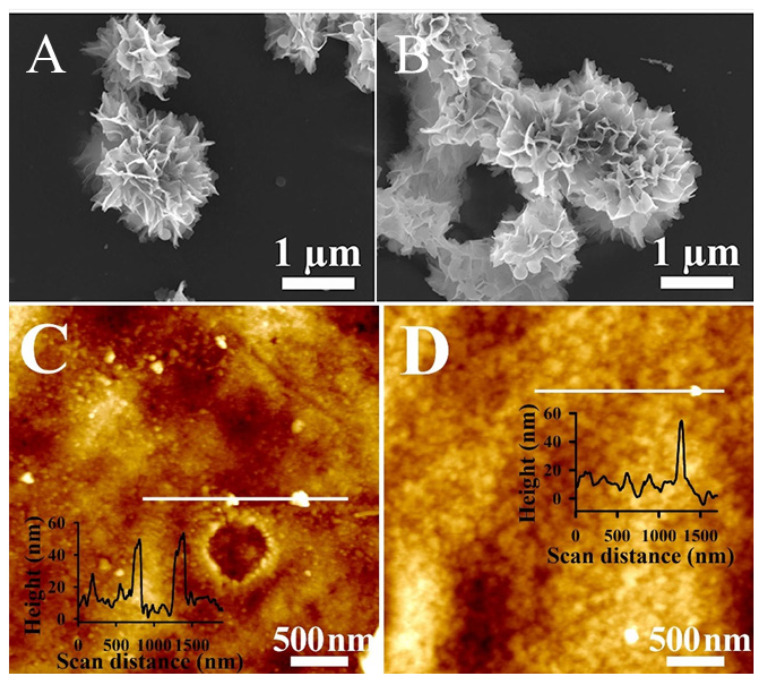
Scanning electron microscopy (**A**,**B**) and atomic force microscopy (**C**,**D**) images of poly(styryl bisphosphonate) (poly(StBP))-6, and poly(StBP)-40 (**D**) films, respectively, where “6” and “40” represent the thickness of the Mayer rod (6 and 40 µm) used to spread the polymer solution. (Reprinted with permission from [62] Copyright (2020), Elsevier).

Cross-linked poly(styryl bisphosphonate) (poly(StBP)) thin coatings (thickness: 163 ± 8 and 175 ± 7 nm) were applied to corona-treated PP films by UV curing for bone tissue engineering applications by Steinmetz et al. [62]. Initially, poly(StBP) nanoparticles were prepared and mixed with poly(ethylene glycol) dimethacrylate and a photo-initiator. Next, they were spread on the PP film and cured using UV radiation. The authors observed that the poly(StBP) nanoparticle-embedded coating induced apatite crystal growth (Figure 5), which resulted from the strong affinity of poly(StBP) toward calcium ions. Furthermore, the coating exhibited durability and optical properties. The authors claimed that this coating method could be useful in various bone tissue engineering applications.

### 5.4. Polydimethylsiloxane (PDMS)

PDMS is a synthetic material that is used extensively in various biomedical tools such as surgical implants, catheters, contact lenses, pacemaker encapsulants, and biosensors, as well as in drug delivery and DNA sequencing owing to its excellent properties, such as bioactivity, greater flexibility, ease of fabrication, oxygen permeability, optical transparency, and low toxicity [63,64]. Furthermore, it can be used as an ideal organ-on-chip substrate to study stem cell behavior. The characteristics of this material make it a suitable candidate for studying cell activities, such as topography, stretching, and mechanical and electrical stimulations for designing materials for tissue engineering applications [63]. However, the interaction of PDMS with cells is limited, which necessitates the modification of its surface characteristics to achieve the desired properties. Similar to PP, plasma treatments can be used to modify the surface of PDMS by the creation of hydroxyl groups.

**Figure 6:**
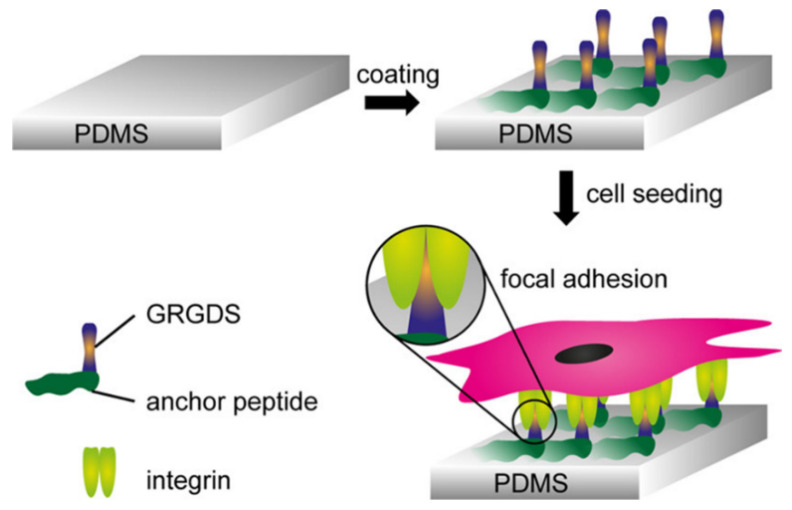
Schematic illustration of polydimethylsiloxane (PDMS) coating using engineered anchor peptides fused to the cell-adhesive peptide sequence (glycine-arginine-glycine-aspartateserine, GRGDS). (Reprinted with permission from [63] Copyright (2019), American Chemical Society).

Gehlen et al. [63] reported a novel, one-step PDMS coating method using engineered anchor peptides fused to a cell-adhesive peptide sequence (glycine-arginine-glycine-aspartateserine). In this method, hydrophobic interactions were used to attach the anchor peptides to the PDMS surface by dipping the PDMS substrates in an anchor peptide solution (Figure 6). Binding performance, cell attachment of fibroblasts, and endothelial cells were studied, and the coating conditions were optimized. The authors claimed that this method employed mild conditions and room temperature, and could be easily used to functionalize biomedical devices with sensitive and complex components.

Ultralow friction was established in an aqueous environment for using PDMS by bonding of a poly(acrylamide–acrylic acid) hydrogel coating on the surface of the substrate [65]. Bonding was achieved through chemical modification of the PDMS surface and successive reaction with acrylic acid moieties. The product reduced friction by two orders of magnitude in an aqueous environment, which had a friction coefficient (µ) as low as 0.003 [65]. A chlorhexidine (CHX)-loaded PDMS-based coating was applied on the surface of a 3D-printed dental polymer to induce surface wettability, microstructure, and antibacterial activity [66]. CHX was encapsulated in silica nanoparticles and added to PDMS to produce an antibacterial coating material. This was coated as a thin film on a 3D printed specimen using oxygen plasma and by subsequent heat treatment. This coating eventually increased the surface hydrophobicity and reduced the irregularities. Furthermore, it notably reduced bacterial colony formation compared to that in the uncoated samples.

**Figure 7:**
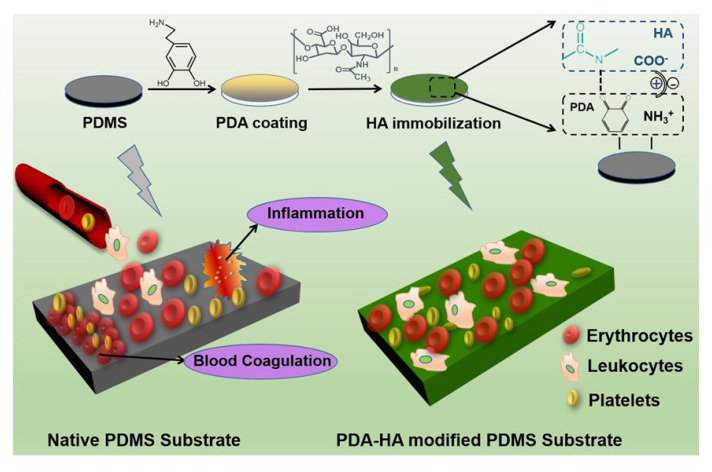
Schematic illustration of polydimethylsiloxane (PDMS) surface modification using hyaluronic acid and polydopamine (HA/PDA) composite coatings. (Reprinted with permission from [67]. Copyright (2017), American Chemical Society).

PDMS with enhanced hemocompatibility was developed for medical implant or device application by modifying the surface using a PDA and hyaluronic acid (HA/PDA) composite [67], as shown in Figure 7. Enhanced hemocompatibility was observed for a particular HA/PDA composition, using which platelet adhesion and activation were reduced, compared to that observed in other combinations of PDMS and HA or PDA coatings. Furthermore, it was observed that along with hemocompatibility, anti-inflammatory effects and cytotoxicity could also be altered by adjusting the HA and PDA composition on the PDMS surface. These advantages can be useful for the development of medical implants and devices.

**Figure 8:**
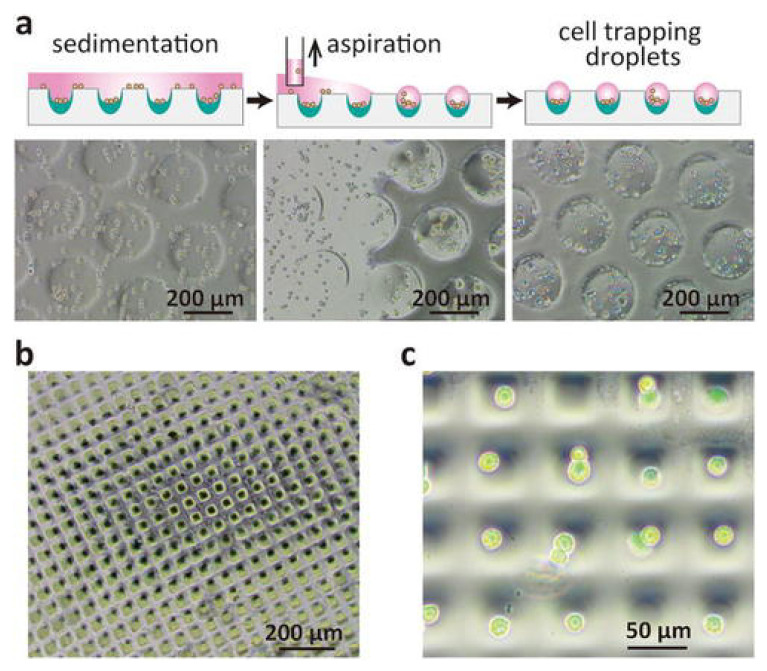
Schematic illustration of the cell trapping mechanism in polydimethylsiloxane (PDMS) microwells and the corresponding micrographs in (**a**) 200 µm and (**b**) 35 µm square microwell arrays. (**c**) Single-cell trapping demonstrated using a combination of bright field microscopy and fluorescence imaging. (Reprinted with permission from [68] Copyright (2018), American Institute of Physics).

Another interesting and single-step surface modification for producing a long-lasting hydrophilic surface was performed using microwell arrays, as reported by Oyama et al. [68]. In this method, a low-energy electron beam (55 kV) was used to irradiate PDMS films in an air-produced silica-like layer with a thickness of 40 µm. This modified surface showed prolonged hydrophilicity for more than 10 months in aqueous medium. These microwells were able to trap cells/single cells and provide stable and promising cell adherent environments (Figure 8). Since no chemical was used in this method for surface modification, the intrinsic bioactivity of PDMS was retained. This notable and promising result revealed that the platform could be interesting in lab-on-chip applications, medical applications, drug screening, and stem cell studies [68].

In another study, Mahmoodi et al. [69] reported the surface modification of PDMS using PTFE coatings to avert the absorption and adhesion of solvents onto PDMS microchannel walls, which can be used for the encapsulation of anti-inflammatory drugs. The results showed that after the coating, the microchannels exhibited super hydrophobicity (140.30°), which effectively prevented the adhesion and absorption of solvents by the drug-loaded nanoparticles. Furthermore, the drug release and encapsulation efficiency were favorably altered by the coatings without any toxicity.

### 5.5. Polyurethane (PU)

Among synthetic polymers used in medical applications, PU is used only a small fraction in spite of its application in various fields. PU coatings have significant uses in various fields, including biomedical applications. In the medical field, it is primarily used to manufacture pacemaker lead coatings, breast implant coatings, and vascular devices. In recent times, PU has garnered significant attention owing to its bioactivity, biodegradability, and adaptive physical and chemical forms. Furthermore, its physical and mechanical characteristics are similar to those of natural tissues [70,71]. PUs consist of alternating hard and soft segments (HS and SS). The SS has an elastomeric character, whereas the HS offers additional strength owing to the hydrogen bond-containing urethane linkages [72,73]. Its biodegradability, physicochemical properties, and other properties can be adjusted by altering the ratio between the SS and HS components, chemical composition, and molar weight of PU in specific applications [70,74]. Joseph et al. reviewed the biomedical applications of PU and its coatings [75].

New biodegradable freestanding PU films were produced without using any catalyst by Barrioni et al. [70]. The authors developed an HS using hexamethylene diisocyanate and glycerol and an SS using poly(caprolactone) triol and low-molecular-weight PEG. A highly homogeneous PU structure with an interconnected network was formed. The deformation at break reached 425.4%, and the elastic modulus and tensile strength were 1.6 MPs, and 3.6 MPa, respectively.

Bacterial resistance PU coatings for medical devices were developed by Roohpour et al. [76]. To inhibit microbial film formation, silver lactate and silver sulfadiazine were capped with the polymer. The silver ions were found to be covalently bonded with PU without affecting its mechanical properties, while an adequate bactericidal effect was exerted even when the silver content was low. This material can be used for developing medical device coatings and associated applications. In another study, the water resistance and bioactivity of PU were improved by the addition of isopropyl myristate, which modified the hydrophobicity of PU [77]. The surface properties of PU changed and its surface energy was reduced. The modified PU exhibited considerably lower water permeability than the silicon packing materials available currently. This may be considered a suitable material for electronic implants. Recently, PUs for medical implants and devices have been prepared by 3D printing, and the latest developments in the 3D printing of PU in biomedical fields have been reported by Griffin et al. [78]. Similarly, a review on bio-based PU for biomedical applications was published by Wendels et al. [79]. Apart from regular coatings, PU/graphene based electrospun nanocomposite fibers were reported to increase the electroconductivity, bioactivity and mechanical properties [80].

## 6. Other Biopolymer Coatings

Apart from the polymers mentioned above, various other biopolymers such as poly lactic acid (PLA), Poly (lactide-co-glycolic) acid (PLGA), Polycaprolactone (PCL), polyethylene (PE) and some natural polymers such as collagen and chitosan are also used for various biomedical applications. PLA is recyclable, hydrophobic aliphatic polyester used for various biomedical applications, such as medical devices, tissue engineering, drug delivery and 3D printed scaffolds. ZnO nanoparticles embedded in PLA was dip coated on Mg alloy (AZ31), which helped to control the degradation and promote antibacterial activity [81]. Incorporation of ZnO in PLA matrix provide control over surface topography and Mg degradation rate. A free-standing PLLA micro-chamber array was developed by dip coat PLLA solution on PDMS stamp followed by drug loading and sealing with pre-coated polymer substrate, for drug delivery application [82]. A low frequency ultrasound trigger the release of drug and in vitro test revealed that the full cargo of drug was completely released in 13 days under physiological condition. It can be used as a smart polymer which can deliver the drug by stimuli.

Another biodegradable polyester is PCL which showed brilliant properties, such as bioactivity, biodegradability and flexible mechanical properties. In biomedical field, PCL can be used for tissue engineering applications, drug delivery and bone graft material. An -g based alloy was coated with PCL or PCL nanocomposite coatings to improve its functionalities, such as osteogenesis, bioactivity and adhesion strength. Kim et al. provide a uniform coating of PCL on Mg screw to improve its osteogenesis [83]. In order to increase the adhesion between Mg and PCL, plasma electrolytic oxidation (PEO) was performed and then PCL was coated by dip coating method. With an optimized coating conditions, thick and dense bone formation was found around the PCL coated screw in rat femur. In another study, PCL/fluorine doped apatite (FHA) composite duplex coatings was performed by dip coating method, on the Mg alloy to improve its biological properties and control the degradation rate of Mg alloy [84]. The bilayer PCL/FHA coatings provide good corrosion resistance and biomineralization formation, which can be used for implant applications. In another study, in order to improve the antifogging and low oxygen barrier of PCL, multilayer coatings of poly(vinyl alcohol) (PVA) and tannic acid (TA) bilayers were used which reduced the oxygen permeability with the presence of 20 bilayers and fogging was controlled with five bilayers [85]. This work opens up a way to design transparent biodegradable coatings with oxygen barrier and antifogging properties for various applications. PCL based PU electrospun microfiber with apatite nanoparticles were prepared to enhance the biological characteristic and shape memory properties. This composite nanofibers showed controlled drug delivery [86]. The addition of apatite with various ratio to determine the shape memory features and 3 wt% of HA showed excellent recovery with short recovery time.

**Figure 9:**
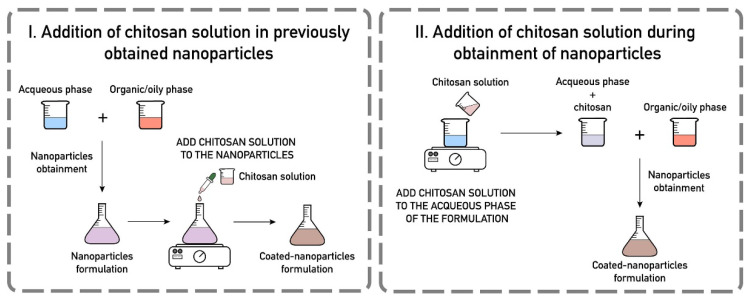
Different approaches of chitosan coatings for nanoparticles. (Reprinted with permission from [90] Copyright (2020), Elsevier).

Natural polymers such as chitosan and collagen were also used as coatings for improve the functionalities of the biomaterials. Various reports are available for the chitosan coatings for biomedical applications. One of the coating method is electrophoretic deposition. Various recent reports provide an overview of this coating method [87,88,89]. Avcu et al. recently reviewed the chitosan based composite coatings for biomedical applications [89]. Very recently, Frank et al. provides a comprehensive review about chitosan coatings on nanoparticles [90]. Chitosan coatings for nanoparticles are carried out in two ways: (i) Initial preparation of nanoparticle and then adding chitosan solution to the nanoparticles, (ii) addition of chitosan during nanoparticle preparation (Figure 9).

Collagen is another natural polymer and is the richest constituent of ECM. As expected, coating a biomaterial with collagen can improve its bioactivity, its ability to form an interface between the host tissue and the implants. Various reports proved that cell proliferation, differentiation and adhesion as well as new tissue formation was improved with the collagen coatings [91,92]. The cell spreading and growth can greatly influenced by the solvent used to prepare the collagen coatings [93]. In order to increase the mesenchymal stem cell (MSCs) adhesion, survival and proliferation collagen coatings were tried and found to be effective [92]. A combination of collagen coatings on chitosan shown to promote cell attachment and distribution [94].

## 7. Biopolymer Coatings on Nanoparticles

In a nanoparticle system, surface optimization is needed for the practical use of nanoparticles in clinical applications. In systematic drug delivery systems, surface functionalization and coatings on nanoparticles can be highly useful for altering the selectivity of nanoparticles in the delivery procedure to produce a system with better targeted drug delivery potential. The choice of the coating material is particularly important in biomedical applications, since in some cases, surface modification may alter the properties of nanoparticles, and consequently, its performance in clinical applications. This is especially true for magnetic nanoparticles (MNPs), as some coatings may change their magnetic properties. In medical applications, it is important to consider parameters, such as bioactivity, toxicity, stability, and support for anchorage of other functional groups, since these coatings play a vital role and can perform multiple functions simultaneously. One of the best tools in this respect is universal polymer coating, which can be applied to a variety of material surfaces and has outstanding prospects for biomedical applications owing to the flexible surface modification process and absence of substrate conditions, such as stiffness and topography [95].

MNPs are used in various applications and have been considered suitable candidates for biological applications in recent years. They are used for magnetic resonance imaging (MRI), diagnostic imaging, magneto-optical based immunoassays, and magnetic hyperthermia induction in tumors [96,97]. To use MNPs in the aforementioned applications, it should be ensured that these do not agglomerate due to colloidal or magnetic forces in a given medium. To overcome these challenges, MNPs should be coated with materials that support solubilization in a medium. Biopolymers can satisfy these requirements owing to their bioactivity and biodegradability [90].

**Figure 10:**
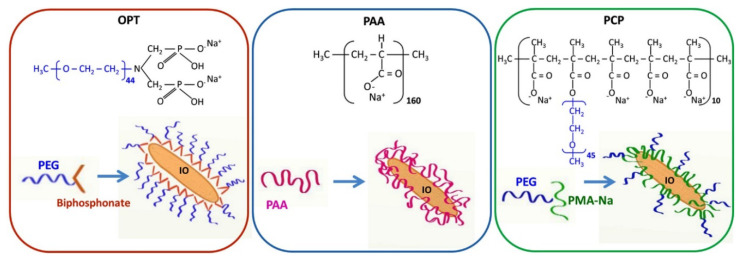
Schematic illustration of iron oxide nanorods coated with linear bisphosphonate−poly(ethylene glycol) (OPT), polyacrylic sodium salt (PAA), and polymethacrylate backbone/PEG side chain comb polymer (PCP). (Reprinted with permission from [97]. Copyright (2018), American Chemical Society).

Iron oxide nano-rods were coated with polymers to improve their colloidal stability in aqueous media by Marins et al. [97]. Three different polymers were coated (Figure 10), and experimental and theoretical evaluations were performed to study the colloidal stability of the nano-rods. Improved nano-rod colloidal stability was observed. The results also showed that this method could be useful for increasing the colloidal stability of rod-like nanoparticles in biomedical applications, where perfect colloidal stability is a necessity. In another recent study, Pereira et al. [96] reported the suitability of chitosan-based hydrogel coatings for magnetic particles for potential drug delivery applications. In the mentioned study, a polyelectrolyte complex of chitosan and *Sterculia striata* gum was used as a coating material. The coating was found to be present on the negatively charged outer layer of the magnetic particles. Unlike the previous study, this study revealed that the Fe_3_O_4_ nanoparticles aggregated and formed large clusters. The results obtained showed that hydrogel-coated Fe_3_O_4_ could be a suitable candidate for drug delivery applications.

Poly(2-(methylsulfinyl) ethyl acrylate) (PMSEA), a highly hydrophilic sulfoxide polymer, was used to modify the surface of the iron oxide nanoparticles to improve their circulation in blood and minimize protein fouling [98]. The particles produced exhibited superior colloidal stability under physiological conditions. Compared to conventional polymer coatings (PEGA), PMSEA coatings provided an enhanced toxicity profile and reduced macrophage and protein interactions. Furthermore, the results indicated the remarkably low fouling properties of PMSEA. Hence, this polymer coating could be of interest in biomedical applications, especially in advanced therapeutic and diagnostic applications.

Chitosan was also used as a thermoresponsive coating on magnetic silica nanoparticles for controlled drug release and magnetic hypothermia applications. Pon-On et al. [99] encapsulated magnetic silica particles within a chitosan-g-N-isopropylacrylamide polymer matrix. This material exhibited superior paramagnetic behavior, and in an alternating magnetic field (AMF), it acted as a heat source with a specific absorption rate of 8.36 Wg^−1^. The drug doxorubicin (DOX) encapsulated in this particle was sensitive to heat and pH; hence, the authors studied the drug release profile using external heat and internal heat produced using an AMF. Approximately at neutral pH (7.4) and under external heating, the drug release was considerably slower. At an acidic pH under internal heating by AMF (4.0), the release was much more rapid. Owing to its in vitro toxicity, the DOX-loaded chitosan-coated magnetic silica nanoparticle exhibited stronger anticancer activity than free DOX. This study demonstrated a promising application of pH/thermos-chemotherapy using an AMF drug delivery system.

Mg_0.5_Co_0.5_Fe_2_O_4_ MNPs were functionalized using chitosan (CS) coatings to enhance the delivery of the loaded anticancer drug 5-fluorouracil (5FU) for the formation of CS-Mg_0.5_Co_0.5_Fe_2_O_4_-5FU [100]. At physiological pH and under acidic conditions, these small, spherical nanocomposites exhibited stability and sustained drug release for more than 48 h. Furthermore, these exhibited improved and selective anticancer activities compared to the free drug. Bioactive polymer-coated paramagnetic (Fe_3_O_4_) MNPs were prepared by Zarouni et al. [101] for DOX delivery in breast cancer treatment. The pH-responsive polymer-coated MNPs were controlled by magnetic and pH values. The size of these nanoparticles was approximately 20 nm, and these had a high encapsulation efficiency. The MNPs exhibited a gate-like action, and rapid release was observed at pH 5.8, which ceased at pH 7.4.

**Figure 11:**
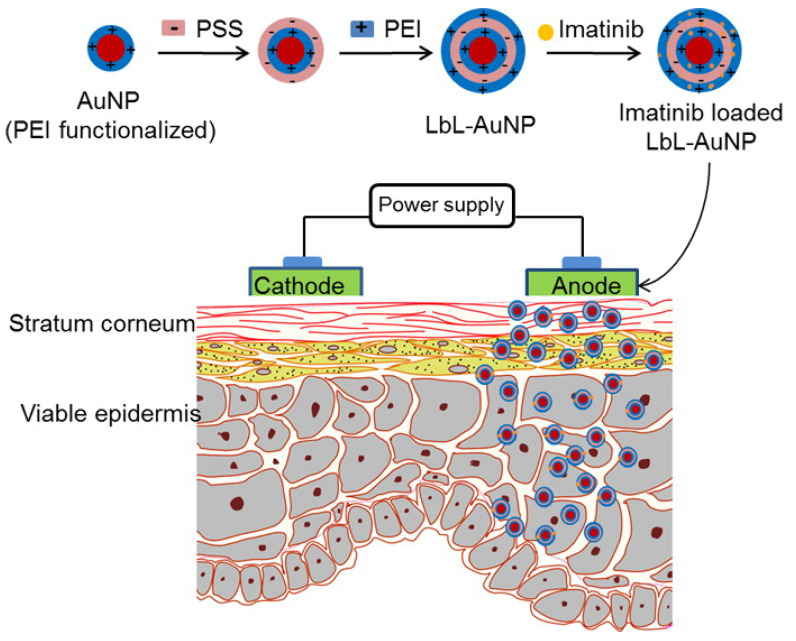
Schematic illustration of imatinib mesylate (IM)-loaded PSS/PEI-AuNPs delivering IM to the layers of skin in melanoma treatment. (Reprinted with permission from [102]. Copyright (2015), American Chemical Society).

For precise targeting and improved biological interactions, the surfaces of gold nanoparticles (AuNPs) have been modified in several studies, and these have become more popular in recent times. Owing to their unique physical and optical properties, AuNPs have garnered significant attention in various biomedical applications. Furthermore, it is easy to functionalize AuNPs using various materials, such as ligands, proteins, DNA, antibodies, and polymeric materials. These properties can be useful in several medical applications. LBL polymer-coated AuNPs have been used as carriers for delivering cancer drugs, such as imatinib mesylate (IM) [102]. AuNPs were prepared and functionalized using polyvinylpyrrolidone and polyethylene imine. Subsequently, they were successively coated with anionic poly(styrenesulfonate) and cationic polyethylene imine. The AgNPs produced were loaded with IM to produce IM-PSS/PEI-AuNPs (Figure 11). The characterization of the nanoparticles formed showed that the loading efficiency was better than that reported in other studies, and an in vitro study revealed that skin penetration was enhanced by 6.2-fold compared to that achieved by passive application. At concentrations greater than 50 μM and 31 μM for gold, and IM, respectively, the IM-PSS/PEI-AuNPs exhibited considerably higher inhibition of cancer cell growth than pure IM. Hence, this nanoparticle may serve as a promising tool for treating melanoma compared to IM alone [102]. Similarly, several research reports have been published on the biomedical applications of polymer-coated AuNPs, including drug delivery, gene therapy, and photothermal therapy and imaging. A review of the latest developments in the use of polymer-coated AuNPs in biomedical applications was published by Fuller et al. [103]. Gold-ferric oxide superparamagnetic nanoparticles (Au–Fe_3_O_4_ NPs) were coated with poly-L-lysine (PLL) to deliver the nanoparticles to cells in breast cancer treatment [104]. Owing to properties such as surface plasmon resonance and super paramagnetic effect, Au can be used as a nano-thermal ablator and MRI contrast agent, respectively. To use PLL-Au–Fe_3_O_4_ NPs in photothermal therapy, these nanoparticles were incubated with breast cancer cells, and their intracellular uptake and cytotoxicity were studied by NIR laser irradiation at 808 nm. The results showed that this material served as an all-in-one theranostic agent and could be used for both diagnostics and photothermal ablation of breast tumors. Table 1 summarizes all the findings from this review.

## 8. Conclusions and Future Prospects

Tissue engineering is an important field in life science research that deals with multidisciplinary and interdisciplinary approaches. In recent times, it has undergone an exponential progress. Polymer-based hybrid materials are garnering attention rapidly, and the development of pioneering coating methods as well as imaging and characterization techniques, along with the availability of new materials and combinations, may help customize biomaterials for specific tissue engineering applications. Among various surface modification methods, surface modification with suitable polymeric materials can be successfully used for practical biomedical applications. However, it is essential to select the appropriate substrate material, polymer, coating method, and most importantly, the area of the material to be used in clinical applications. Hence, it is important to understand the underlying mechanism, and preferable to use theoretical strategies for the development of such coatings. Similarly, in a nanoparticle system, nanoparticles can be used as promising candidates for biomedical applications; however, there are multiple parameters to be considered. Polymer coatings on these materials can help address multiple issues, such as immediate adsorption by proteins and uptake by macrophages. Various nanoparticles that can be modified using different polymer coating methods and have a high potential for use in biomedical applications have been developed and tested. Most of the results discussed above were reported from laboratory studies, and the development of coatings and complicated structures for clinical applications on a large scale is yet to be implemented. The advancements in this field have paved the way for the development of stealth polymer coatings with multifunctional attributes for addressing common challenges experienced in tissue engineering. Compared to other industrial applications, strong evidence of the optimal performance of biomedical coatings in biological environments is necessary before there practical application can be approved in clinical settings. This continues to pose a challenge to advancements in this field, and qualitative research and development are warranted.

## Figures and Tables

**Table 1:** Summary of the polymer coatings for biomedical applications.

Coatings Material	Coating Method	Substrate/Nanoparticle	Applications Area	Refs.
poly(2-methyl-2-oxazoline) (PMOXA)	Electrochemical non brush bionic coating	316L stainless steel	bioactivity, antifouling properties, prevent late stent thrombosis and in-stent restenosis	[15]
Polytetrafluoroethylene	PEO coating	magnesium alloy MA8	protective and antifriction properties	[16]
hydroxyapatite- polytetrafluoroethylene	PEO coating	Mg–Mn–Ce alloys	corrosion resistance and impart bioactivity	[17]
1. CaP coating 2. polylactic acid	immersion dip coating	magnesium	corrosion resistance and elongation of degradation time	[18]
phosphate/collagen (CaP/Col) composite coatings	Chemical conversion and dip coating	Magnesium alloys	corrosion resistance and inducing bioactivity	[19]
PVDF	Spin coating	Free standing ZnO grown film	wearable and wireless pressure sensor for heart rate monitoring	[31]
R-GO/P(VDF-TrFE)	liquid phase blending Spin coating	Flexible and glass substrate	flexible, optically transparent, and highly responsive temperature sensor	[40]
polyaniline-coated PVDF	Electrospinning in situ conversion	Aluminum foil	Human health monitoring	[45]
PVDF-nanosilica	Electrospinning	Aluminum foil	Increased piezoelectric property for biomedical application	[46]
PVDF/conducting polymer	Electrospinning	aluminum foil	Electrical conductivity and bioactivity	[47]
P(VDF-TrFE)	Electrospinning	aluminum foil	implanted energy harvester, bioactivity	[48]
PMMA	Alkali activation and surface-initiated atom transfer radical polymerization	Titanium	hybrid prosthesis, bioactivity	[52,53]
methacrylate	modelling	Titanium	less susceptible biofilm formation coating, bioactivity	[54]
PMMA/PDDA	spin coating or casting and drying	Si, glass, or polystyrene sheets	Antimicrobial coating	[55]
PMMA/AgNPs-CS	immersion method	Soft rubber	Antimicrobial coating	[56]
PDA	cold oxygen plasma	PP hernia mesh	drug absorption and longer release, antibacterial properties	[58]
PVP:PEGDA	Cross-linking	PP	hydrophilicity and bioactivity	[61]
Poly(StBP)	Spreading and curing with UV	PP	Bone tissue engineering	[62]
cell-adhesive peptide	Dip coating	PDMS	functionalize biomedical devices with sensitive and complex components	[63]
poly(acrylamide–acrylic acid)	Chemical bonding	PDMS	Ultralow friction coatings	[65]
chlorhexidine (CHX)-loaded PDMS	oxygen plasma and heat treatment	3D-printed dental polymer	induce surface wettability, microstructure, and antibacterial activity	[66]
PDA and hyaluronic acid	drop casting	PDMS	Hemocompatible medical device and implant	[67]
PDMS	low-energy electron beam irradiation	PDMS	long-lasting hydrophilic surface	[68]
PTFE	Printing/Solution injection and curing	PDMS	To encapsulate anti-inflammatory drugs, super hydrophobicity	[69]
PU	casting	Freestanding films	Biodegradable material for biomedical application	[70]
PU/Ag	end-capped with functional groups	freestanding	developing medical device coatings and associated applications	[76]
Isopropyl Myristate	casting	PU	bioactivity and low water permeability	[77]
PU/graphene	electrospinning	Aluminum foil	electroconductivity, bioactivity and mechanical properties	[80]
PLA/ZnO	Dip coating	Mg alloy (AZ31)	Reduced Mg degradation rate	[81]
PLLA	Dip coating	PDMS stamp	Drug delivery application	[82]
PCL	PEO and dip coating	Mg screw	Bone forming ability and osteogenesis	[83]
PCL/FHA composite duplex coating	Dip coatings	Mg alloy	bioactivity and controlled Mg degradation	[84]
PCL/PU/apatite	Electrospinning	Aluminum foil	controlled drug delivery	[86]
Different polymer coatings	Polymer adsorption	Iron oxide nanorods	perfectly stabile colloidal nanoparticle for medical application	[97]
PMSEA	RAFT polymerization	iron oxide nanoparticles	extended blood circulation time and reduced accumulation	[90]
Chitosan	encapsulation	magnetic silica nanoparticles	pH/thermos-chemotherapy using an AMF drug delivery system	[99]
Chitosan	adsorption	Mg_0.5_Co_0.5_Fe_2_O_4_	Drug delivery	[100]
Chitosan -PMAA	in situ polymerization	Fe_3_O_4_ MNPs	DOX delivery in breast cancer treatment	[101]
PSS/PEI	LBL	AuNPs	Drug delivery to the layers of skin in melanoma treatment	[102]
